# Biomimicry in the Context of Stabilised Porous Clays

**DOI:** 10.3390/biomimetics10050290

**Published:** 2025-05-05

**Authors:** Arya Assadi-Langroudi

**Affiliations:** Engineering Division, University of East London, London E16 2RD, UK; a.assadilangroudi@uel.ac.uk

**Keywords:** nature-inspired, porous, loose, interparticle, small-strain, biopolymer

## Abstract

This study explores the etymological roots of nature and nature-inspired design within the context of soil stabilisation. It outlines Aristotle’s doctrine of hylomorphism and applies these concepts to develop a pathway for the stabilisation of clays within their original porous or looser structure through interparticle modifications. A biopolymer is introduced to a base clay thorough a procedure that imitates forms, matter, generative processes, and functions of arbuscular mycorrhizal (AM) fungi. For the first time, the void ratio was progressively increased from 0.50 to 0.70, and the air ratio from 0.15 to 0.33, reflecting a systematic transition from a denser to a looser packing state. A 20% increase in shear wave velocity indicated enhanced interparticle engagement following treatment. This reinforcement effect contributed to the preservation of stiffness and residual strength, despite a 120% increase in air ratio and a 63% reduction in degree of saturation, alongside a modest improvement in unconfined compressive strength. The findings presented here mark a departure from both conventional and emerging stabilisation techniques, enabling engineered soil to remain porous, to loosen with time, and to continue delivering engineering and ecological services.

## 1. The Philosophy

Nature encompasses both living and non-living entities, as well as the complex systems that emerge from them, such as ecosystems. An entity can be classified as part of nature only if it can be perceived through physical, biological, or chemical observation, and be capable of self-production [1]. In living beings, self-production serves the purpose of instinctual survival and communication. Flora and fauna populations, as well as human societies, self-produce to sustain themselves. The concept can be extended to non-living entities. For example, Assadi-Langroudi et al. [2] demonstrated the natural abundance and deficiency of certain universal quartz particle sizes. They examined particle breakage in erosive environments, showing that some particle sizes continue to crush indefinitely, allowing others to maintain their size. The surviving particles either remain intact, supported by a matrix of finer particles, or result from the aggregation of fine fragments. This trait can be interpreted as a form of self-production for instinctual survival.

For an entity to be considered part of nature, it must not only self-construct but also self-delimit—that is, it must have the capacity to form and maintain boundaries. In other words, for a material to be deemed natural, it must be distinct from its surroundings through some form of limit or interface that is intrinsic to its being. Water, for example, is defined by the presence of a meniscus. In humans, the boundary is the skin. Natural entities may coexist and remain in some form of mutual contact. For instance, mycelium grows contiguously alongside tree roots, with the boundary defined by the interlocking or interparticle forces between mycelium and root molecules.

According to Aristotle’s doctrine of hylomorphism, every natural being comprises four components. First is matter, the material that constitutes the entity. Second is form, the structure of that material and the organisation of its various domains. Third is function, referring to the services the system provides. Fourth are the processes, the mechanisms by which the system comes into being. Nature-inspired design, or biomimicry, involves the selective adoption, understanding, and imitation of these traits. While these four components are distinct, they are fundamentally interconnected.

Nature-inspired or nature-based design emerges from viewing nature as a model, measure, and mentor. It represents an epochal shift—a revolution—rather than a mere repetition of natural technologies. Instead, it is a creative reinterpretation of technē [3]. Nature-inspired design involves the observation, understanding, and imitation of the conditions necessary for an entity to self-produce and self-delimit. As a model, by imitating the aesthetic qualities of a natural material—such as size, shape, texture, and fabric—one can regenerate specific functions. As a measure, a design that aligns with nature is inherently valid, as nature itself embodies the norm or standard. As a mentor, nature must be studied, understood, and applied in the context of techniques and mechanisms.

A natural entity consists of matter, form, functions, and generative processes. Given the interdependency of these components, constructing a biomimetic model follows several key steps: to identify constituting systems that may act as boundaries and offer imitable traits, to select elements within the system—whether matter, form, function, or process—either individually or in combination, and to determine the level of abstraction, refining the extent to which these elements are translated into design.

## 2. The Biomimetic Approach

### 2.1. The Problem

The problem addressed in this paper is threefold. Firstly, loose silts and low-plasticity clays tend to deform excessively under loading, wetting, or a combination of both [4]. Secondly, almost all mechanical, chemical, and hydraulic soil stabilisation methods involve densification. This reduction in void spaces compromises the ecosystem that is core to the growth of subsurface micro-organisms and the movement of fluids. Thirdly, the decreasing availability of fly ash [5] and ground granulated blast furnace slag―most globally common alternatives to Ca^2+^-based binders―has incentivised research into and the use of alkali-activated geopolymers [6,7].

### 2.2. The Natural System

Soil is a natural, porous, grand system that is made up of solid particles sitting against one another and surrounded by void spaces. The pores are partially filled with water and partially with air. Mycorrhizal fungi, specifically arbuscular mycorrhizal (AM) fungi (*phylum Glomeromycota*), inhabit soil and interact with plant roots.

**Figure 1:**
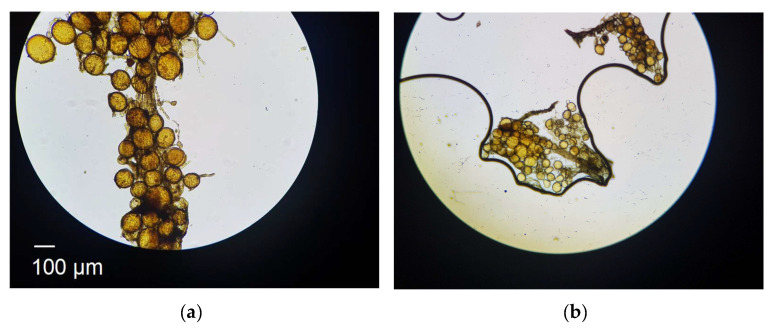
Photomicrograph of inactive root blade colonised by Arbuscular mycorrhizal fungi at (**a**) low-magnification, (**b**) high magnification.

Figure 1a shows the initial colonisation of the root by AM fungal spores. The round structures are the reproductive structures of AM fungi known as spores. When spores settle on and colonise the root lamina [8], they germinate, creating hyphal networks―thread-like structures that penetrate the soil. The filamentous structures in Figure 1a are the hyphae connecting the spores. Figure 1a illustrates the growth of hyphae from spores. The growth of these hyphae pushes soil particles apart, expanding the pore structure and increasing overall porosity. This mechanical rearrangement of soil particles modifies both the infiltration characteristics and the hydraulic conductivity of the soil [9]. As hyphae infiltrate the soil, they not only create additional voids (thus increasing air volume and porosity) but also secrete exudates, which act as natural binding agents, linking soil particles together. This biotic binding process enhances the structural integrity of the soil while reducing the overall volume of bulk water as hyphae consume it for growth. The result is a more cohesive soil structure with improved aeration, higher porosity, and increased resilience against erosion [10,11].

Within the context of biomimicry, the soil–fungi is a grand natural system. It constitutes two local systems, soil (as an assembly of particles) and fungi. The fourfold Aristotelian constituents are as follows: (a) The matter, which includes solid particles and hyphal threads at the macro-scale. At the meso-scale, the matter includes solid particles, fungal spores, and root lamina or an outer layer. (b) The form, which encompasses randomly packed spherical spores that accumulate at throat sites of the root lamina. The form also includes the needle-like threads that eventually extend through the soil’s pore network. (c) The generative processes, which include the formation of spores, their absorption of pore water (thereby decreasing soil water content), and their transformation to hyphal threads. (d) The function, where spores and then hyphal threads push soil particles apart, increase porosity, decrease the degree of saturation, increase air void content, and increase overall strength.

### 2.3. The Abstraction

The principal aim is to adjust the ratio of voids to solids in a base soil to 50 and 70%, and to achieve elevated levels of strength and stiffness across the strain spectrum at a lower-than-plastic-limit working water content.

A base clayey silt soil is mixed with pressmud (PM), a polysaccharide-rich residue of sugar refinery [12]. A sodium silicate solution activator is used to enable geopolymerisation [13]. Carbon dioxide is flushed in the curing chamber in controlled proportions over a period of 21 days. Specimens are allowed to air-dry over the curing time.

**Figure 2:**
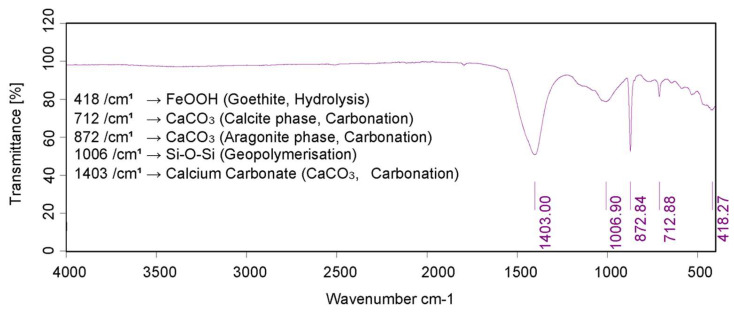
IR spectra for post-treatment samples.

**Figure 3:**
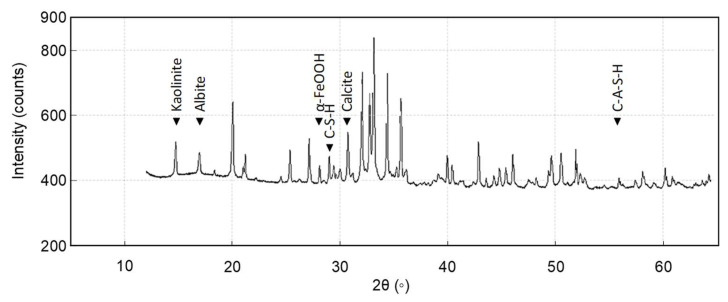
X-ray diffraction spectra for post-treatment samples.

**Figure 4:**
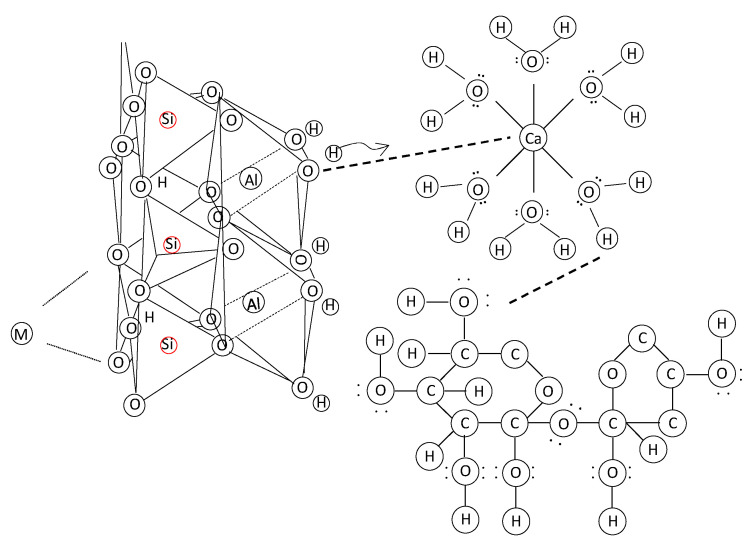
Lewis structure of deprotonated clay following alkali activation, hexaaquacalcium ion from hydration of calcium oxide of pressmud, and organic molecules.

Initially, hydration of calcium oxide (CaO) from the PM forms calcium hydroxide Ca(OH)_2_, which further hydrates to hexaaquacalcium(II) ions or [Ca(H_2_O)_6_]^2+^. The hydration leads to the formation of a complex, honeycomb-like structure resembling the spores of mycelium (see Figure 1). Concurrently, sodium silicate undergoes hydrolysis, enabling deprotonation of the clay fraction and the formation of a sodium aluminosilicate geopolymer, which acts as a binder. Carbonation reactions follow. Ca(OH)_2_ reacts with CO_2_ to form precipitated calcium carbonate (CaCO_3_). In Figure 2, the 1403 cm^−1^ peak is associated with the precipitated carbonate. The peaks at 872 and 712 cm^−1^ are representative of Aragonite and Calcite polymorphs. Precipitation and progressive evolution of Aragonites to Calcites are discussed in detail in Assadi-Langroudi et al. [14], which interested readers are encouraged to read and expand. The presence of ferric oxides (Fe_2_O_3_) within the PM promotes hydrolysis and the formation of goethite (α-FeOOH)―with a peak at 2θ=28° on the diffractogram in Figure 3, which acidifies the system and competes for CO_2_ reactivity, delaying carbonation by forming a protective layer around Ca(OH)_2_. The 418 cm^−1^ band reaffirms the presence of goethite. Pozzolanic reactions take place as silicate and aluminate species interact with calcium, forming calcium silicate hydrate (C-S-H) and calcium aluminate hydrate (C-A-H) gels, which contribute to strength development. Cautiously put, the broad peak around 29°2θ and the hump at 35°2θ on the diffractogram in Figure 3 represent the C-S-H and C-A-H phases. Organic molecules in the PM, particularly polysaccharides, act as nucleation sites or adsorption agents for C-S-H, influencing gel structure and hydration pathways. Water bridging occurs through hydrogen bonding between [Ca(H_2_O)_6_]^2+^ and ether groups in organic molecules, facilitating a controlled growth of cementing gels while regulating porosity. Figure 4 illustrates the nucleation of gels among the trio of the ether group, deprotonated clay, and hexaaquacalcium(II) ions. In Figure 4, the Ca^2+^ ion bridges with Si-O groups and H_2_O ligands, indicating C-S-H formation. Despite the pozzolanic potential, limited alkalinity due to the low A/PM ratio and progressive water retention by organics result in incomplete geopolymerisation, restricting the extent of strength gain and carbonation.

## 3. Materials and Methods

### 3.1. Materials

The base soil was collected from a Trent Riverbank fissured, red brown, and friable Mercia Mudstone exposure near Clifton Manor at coordinates E454032 N334910. The soil is a low-plasticity clay (plasticity index of 11%), comprising a 73 wt.% of sub-63μm fraction and 0.5% of organic matter. The Cation exchange capacity is 20 meq/100 g and the specific surface area (by moisture adsorption) is 49 m^2^·g^−1^. An X-ray fluorescence scan showed 66% of SiO_2_, 28% of Al_2_O_3_, 5% of CaO, and lower contents of Fe_2_O_3_, SO_3_, and P_2_O_5_. An X-ray diffraction scan showed 65% quartz, 17% gypsum, 6.5% k-feldspar, and 4% illite. Soil was air-dried, screened through a 2 mm sieve, crushed in controlled masses using an RM100 Retsch mortar mill, and screened again through a 250 μm sieve. In controlled proportions, the dry screened material was combined with 25% dry mass of pressmud. Deionised water and a dilute sodium silicate solution (1.6 g of water per 1 g of solids) were used to adjust the water content. The solution, the alkali activator, was added to the batch at a concentration of 22% by the dry mass of pressmud―equivalent to approximately 2% of the total mass of the mix. The wet mixtures were statically compacted in eight sub-layers using a Harvard miniature compactor to produce specimens with a diameter of 33.34 mm and a height of 71.52 mm. The degree of saturation, void ratio, and air void content were adjusted. Specimens were cured for 21 days in a sealed chamber at a constant temperature of 21 ± 0.5 °C. During curing, the specimens were exposed to carbon dioxide gas at 1 bar atmospheric pressure for 30 min every 12 h. Table 1 lists the specimen diet and principal properties. In Table 1, *e* is the void ratio (the ratio of the volume of voids to the volume of solids), *S* represents the degree of saturation (the ratio of the volume of water in voids to the volume of voids), and *A* indicates the air void content (the ratio of the volume of air to the total soil volume).

### 3.2. Setups

The testing system comprised a cylindrical test specimen in a strain-rate-controlled loading frame. Specimens were subjected to unconfined compression at a 0.5 mm·min^−1^ rate. Shear wave velocity measurements were conducted using a bender element suite on an unconfined unsaturated specimen. The transmitter was located at the base plate. The receiving transducer was positioned at a 71.52 mm distance from the bottom. The transmitter was excited with a sine pulse of 20 V magnitude. For each specimen, a range of input frequencies between 100 Hz and 10 kHz was tested to determine the ideal frequency that provides greater amplification in the received signals, that is, the clearest output signals.

## 4. Results

**Figure 5:**
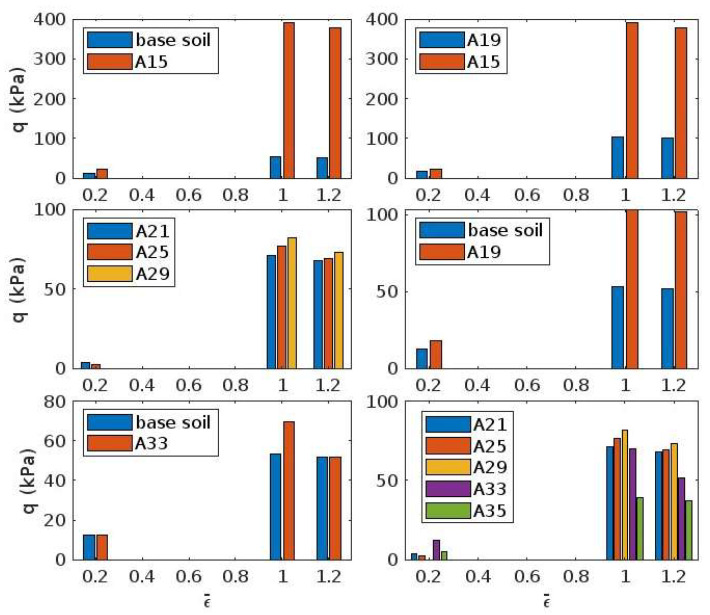
Unconfined compressive stress at various normalised strains, $\bar{\varepsilon }=\varepsilon /\varepsilon_{p}$ and $\varepsilon_{p}$. Note, A denotes air void content in percent.

The base soil comprises 4.8% clay-sized particles by weight and is categorised as CL under the Unified Soil Classification System. The plastic limit and liquid limit are 18% and 29%, respectively. At a void ratio of 0.5 and a degree of saturation of 55%, the unconfined compressive strength is 53 kPa. Figure 5 present the results obtained from a series of unconfined compressive strength tests carried out on the treated base soil. On the diagrams, *q* refers to the deviator stress or compressive stress applied to the soil in the absence of lateral (sideways) pressure. The parameter ε refers to axial strain (the ratio of the vertical displacement to the original height of soil specimen). When deviator stress peaks, ε becomes $\varepsilon_{p}$, or the strain at peak.

In Figure 5, $\varepsilon /\varepsilon_{p}=0.2$ is an indication of material stiffness. The peak strength occurs at $\varepsilon /\varepsilon_{p}=1.0$. Residual strength can also be cautiously interpreted from stress at $\varepsilon /\varepsilon_{p}=1.2$. For the pair base–A15, polymerisation and carbonation clearly demonstrate their effectiveness, as both peak and residual strength improve. Comparing the behaviour of the base and A33 specimens, one notices that a concurrent increase in void ratio and air void content (hence drop in degree of saturation) does not necessarily weaken the soil, provided polymerisation between particles occurs. Maintaining the degree of saturation constant, an increase in void ratio and polymerisation at the contact level has a similar effect—see the sample pair base–A19. However, for soils equally treated with geopolymers, an induced increase in void ratio at constant degree of saturation would jargonise the strength—see sample pair A15–A19. The compressive strength only falls below that of the base soil when the air void content is increased beyond a threshold.

This strength trend is consistent with the following mechanisms: A15 and A19, with higher degrees of saturation (≥0.55), support extensive hydration and early-stage geopolymerisation. As S drops (A21–A29), strength remains comparatively high due to continued formation of C-A-H and C-S-H gels, supported by some residual water and alkali availability. However, at A33 and A35 (S ≤ 0.2), water becomes insufficient for complete hydration of Ca(OH)_2_ and activation of silicates and aluminates, leading to incomplete geopolymerisation. The presence of ferric oxides increasingly competes for CO_2_, delaying or suppressing carbonation. These effects collectively explain the fall in strength, particularly at A35, despite continued porosity and apparent treatment.

The threshold was around A = 33% in the experiments reported here. This suggests a threshold degree of saturation, between 15% and 20%, below which polymerisation and cementation have minimal effect. Observing samples A21-A25-A29, variations in air void content between 20% and 30% appear to have little impact. Comparing the larger A21-A25-A29-A33-A35 group of samples, the control of polymerisation seems to diminish as the degree of saturation drops from 40% to 30%. This can be an indication of ferric oxides becoming dominant controllers. Peak strength decreases from 70–80 kPa to 40–70 kPa as the initial degree of saturation progressively decreases from 50% to 15%. This reduction is directly associated with a deficiency of bulk water necessary for hydration processes.

**Figure 6:**
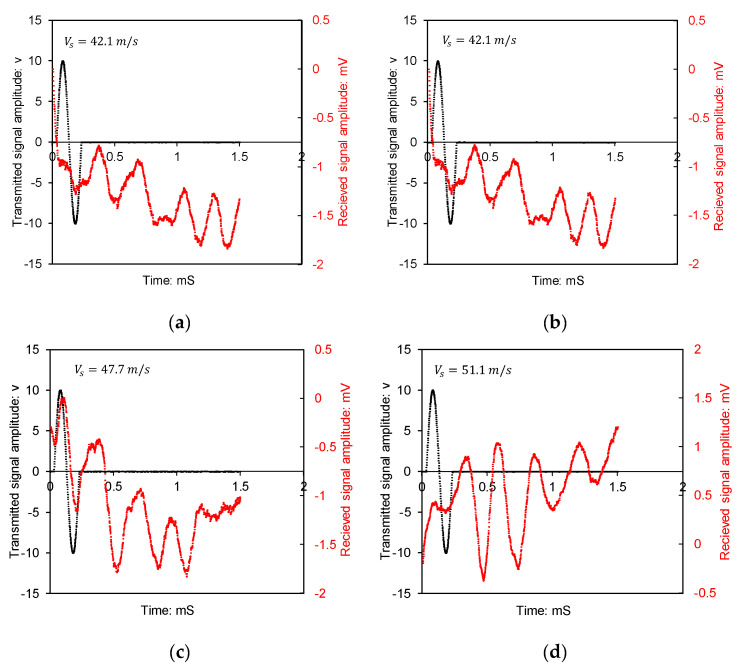
Time domain plot of acoustic emission tests (bender element): (**a**) base soil, (**b**) sample A15, (**c**) sample A21, (**d**) sample A29. Note, $V_{s}$ denotes shear wave velocity.

Figure 6a–d show the time history of BE transmitter and receiver signals―amplitude versus time. The shear wave begins to ascend since the polarity of the input and output signals is positive. The shear wave (S-wave) velocity is the ratio of effective travel distance (tip to tip for transducers) to travel time.

Shear wave velocities were estimated from the bender element test results by visually identifying the arrival time of the first distinguishable shear wave in each time domain waveform. The interpretation method followed the tip-to-tip distance approach, as discussed by Dyvik and Madshus [15]. In Figure 6, the horizontal axis is elapsed time in milliseconds. The arrival time was determined by observing the point at which the waveform deviated from its initial flat or noisy region into a recognisable shear wave pattern. Using the known distance between the transmitting and receiving bender elements, the shear wave velocity was calculated.

The findings demonstrate a progressive increase in shear wave velocity with the increase in air void content and reflect the influence of interparticle cementation in porous structures on interparticle stiffness. For specimens with air void ratios ranging from 15% to 29%, the estimated small-strain shear wave velocities fell within the expected range for partially saturated, weakly cemented silts, aligning with the values reported by Gu et al. [16] and White [17]. Cementation led to a stiffened response at small strains even in soils with relatively loose packing. The similarity in wave arrival shapes across multiple specimens supports the repeatability of the bonding mechanism imparted by the treatment.

## 5. Conclusions

This study introduces a new way to stabilise soil by taking inspiration from nature and ancient philosophy. Using Aristotle’s idea of hylomorphism—which looks at matter, form, function, and process—this study outlines a method that increases the soil’s void-to-solid ratio from 50% to 70% and air-to-void ratio from 15% to 33%, while improving strength and stiffness across both elastic and plastic ranges. Inspired by the behaviour of arbuscular mycorrhizal (AM) fungi, the treatment creates a network within the soil by activating and carbonating waste from sugar refineries. This mimics the way fungal threads bind soil in nature. Unlike traditional methods that rely on compacting the soil, this approach keeps the soil loose and porous. The experiments—albeit at pilot scale—show that the treated soils develop good stiffness and strength, even with high porosity. The shear wave velocities confirm strong interparticle bonding, and compressive strength tests show that the improvements come from bonding between particles rather than densification. The method remains effective up to about 30–33% air void content. A strength threshold was identified at specimens with 33% air void content. The threshold is related to the combined effects of reduced water content, limited alkalinity, and the progressive dominance of ferric oxides. As the degree of saturation drops below approximately 20%, the availability of bulk water becomes insufficient to sustain full hydration of calcium compounds and effective pozzolanic reactions. This limitation restricts the formation of gels and inhibits the mobility of ions necessary for geopolymerisation and carbonation. Simultaneously, the water deficiency encourages the formation of passivating layers of ferric oxides, which compete for CO_2_ and delay carbonation.

## Figures and Tables

**Table 1:** Test specimen diet.

ID	PM wt.%	A/PM †	e	S	A
base	0	-	0.5	0.55	0.15
A15	25	0.22	0.5	0.55	0.15
A19	25	0.22	0.7	0.55	0.19
A21	25	0.22	0.7	0.5	0.21
A25	25	0.22	0.7	0.4	0.25
A29	25	0.22	0.7	0.3	0.29
A33	25	0.22	0.7	0.2	0.33
A35	25	0.22	0.7	0.15	0.35

† A = alkali activator content by mass; PM = pressmud content by mass.

## Data Availability

The data can be made available upon a direct request emailed to the author.

## Abbreviations

The following abbreviations are used in this manuscript:

BE	Bender Element
$V_{s}$	Shear Wave Velocity
$G_{max}$	Small-Strain Shear Modulus
PM	Pressmud
AM	Arbuscular Mycorrhizal
